# Efficacy and Cost Analysis of Food-Based Interventions to Treat Children With Undernutrition: A Systematic Review

**DOI:** 10.21315/mjms-01-2026-016

**Published:** 2026-04-30

**Authors:** Siti Helmyati, Mutiara Tirta Prabandari Lintang Kusuma, Muhammad Hafizh Hariawan, Widjaja Lukito, Yuliana Novita Rachmawati, Gifani Rosilia, Renita Renita, Cut Alima Syarifa

**Affiliations:** 1 Department of Nutrition and Health, Faculty of Medicine, Public Health, and Nursing, Universitas Gadjah Mada, Yogyakarta, Indonesia; 2 Center for Health and Human Nutrition, Faculty of Medicine, Public Health, and Nursing, Universitas Gadjah Mada, Yogyakarta, Indonesia; 3 Nutrition Study Programme, Faculty of Health Sciences, Universitas ‘Aisyiyah Yogyakarta, Yogyakarta, Indonesia; 4 College of Clinical Nutrition, Jakarta, Indonesia; 5 Human Nutrition Research Center, Indonesia Medical Education and Research Institute, Faculty of Medicine, Universitas Indonesia, Jakarta, Indonesia; 6 Department of Nutrition Science, Faculty of Public Health, Universitas Ahmad Dahlan, Yogyakarta, Indonesia

**Keywords:** food for special medical purposes, undernutrition, child nutrition, child growth, cost analysis

## Abstract

**Background:**

Mitigating malnutrition is one of the Sustainable Development Goals that countries commit to achieve. This study aimed to determine the effects of food for special medical purposes (FSMPs) on nutrition status and other outcomes on children under five with undernutrition.

**Methods:**

This review summarised the findings of 15 articles. The articles were experimental, compared intervention with a control group, published between 2012 and 2022, and directly or indirectly measured children’s nutrition status. The risk of bias and quality of the articles were assessed using the Joanna Briggs Institute critical appraisal tool.

**Results:**

Three types of FSMPs were identified as treatment for children with malnutrition. Ready-to-use supplementary foods improved child growth in terms of weight gain. Ready-to-use therapeutic foods mostly resulted in higher recovery rates. Oral nutrition supplements yielded significant increases in body weight and height. However, the economic value of administering FSMPs on children with malnutrition was not determined because of data scarcity.

**Conclusion:**

FSMP provision to treat malnutrition in children resulted in better growth outcomes. However, further studies are needed to measure the health and economic effects of FSMPs.

## Introduction

Undernutrition is the main cause of recurrent and severe infections, delayed recovery, and increased mortality risk from common illnesses among children under five. It is caused by prolonged lack of nutrients and intricate interaction of generational and environmental factors (1, 2). Childhood undernutrition negatively affects individuals and communities by increasing morbidity and mortality risks, weakening immunity, impairing motor development, and reducing productivity in adulthood (3–5). Undernutrition reduces academic capacity and adult productivity, hindering economic growth and perpetuating poverty at the community level (6). It also increases vulnerability to non-communicable diseases in later life, significantly raising national health expenditures and costing economies an estimated 2% to 3% of GDP in highly affected countries (6).

Nutrition interventions for malnutrition include specifically formulated therapeutic foods. Dietary management of moderate acute malnutrition (MAM) can include nutrient-dense, locally sourced foods and products, such as ready-to-use therapeutic foods (RUTFs), ready-to-use supplementary foods (RUSFs), and lipid-based nutrient supplements. Dietary management in severe acute malnutrition (SAM) involves therapeutic foods, including F75 milk during stabilisation, F-100 milk during rehabilitation, and RUTFs (7). Additionally, oral nutrition supplements (ONS) can be administered to support patients with malnutrition (8). These products are categorised as foods for special medical purposes (FSMPs); defined as specially formulated and processed foods prescribed for individuals with limited capacity to consume, digest, absorb, or metabolise regular foods or nutrients; intended for total or partial feeding; and used only under clinical supervision (9).

Nutrition interventions to address malnutrition can be specific or sensitive. Both approaches benefit the country, as nutritional improvements can strengthen and promote economic growth (10). According to the Copenhagen Consensus, nutrition interventions are among the most crucial and cost-effective means of advancing human well-being (10). The efficiency of nutrition interventions can be assessed by comparing costs and outcomes, with macroeconomic evaluations highlighting potential societal benefits. Therefore, a systematic review is needed to evaluate the efficiency and economic value of FSMPs in addition to their efficacy in treating malnutrition. Findings could assist in the decision-making process regarding FSMP provision to address malnutrition in children.

## Methods

This systematic review adhered to the Preferred Reporting Items for Systematic Reviews and Meta-Analyses guidelines and has been registered on the Prospective Register of Systematic Reviews (Number CRD42023387805).

### Research Strategy

This review focused on interventions for children with malnutrition using FSMPs. To obtain relevant studies, a research strategy was developed based on the population, intervention, comparison, and outcome (PICO) terms as follows:

- Population: undernourished children in any country
- Intervention: FSMPs
- Comparison: non–FSMP-based intervention
- Outcome: nutrition status and other outcomes directly or indirectly related to undernutrition

Literature searches were conducted on PubMed, EBSCO, and ProQuest. To form the research query, keywords and terms were carefully selected according to the PICO terms and adjusted to the Medical Subject Headings. These queries were used to search the PubMed database: ((((“Diet Therapy”) OR “Dietary Supplements”) OR “Foods, Specialized”)) AND “Malnutrition”) AND “Child, Preschool.” These queries were used on EBSCO and ProQuest: (“undernutrition” OR “malnourishment”) AND “children” AND (“dietary supplements” OR “foods, specialized” OR “food supplements”).

### Inclusion and Exclusion Criteria

Studies included in the analysis had to specifically evaluate the effect of FSMPs on children under five at risk of failure to thrive, moderate malnutrition, or severe malnutrition. Studies of older children were also selected if the analysis included children under five. Studies were original articles employing an experimental study design with a comparison group. Conference abstracts, case reports, review articles, correspondences, and editorials were excluded. Studies without available full texts were also excluded. Our search was restricted to articles written in English or Indonesian and published between 2012 and 2022.

### Article Screening and Extraction

Articles collected from the databases were processed using Rayyan.ai. Before screening, duplicates were eliminated. Two authors (YNR and MHH) separately screened and assessed the articles by titles, abstracts, and/or full texts and categorised them based on the research strategy and inclusion and exclusion criteria. Dissenting opinions regarding the inclusion of articles were settled by the third reviewer (MTPLK).

Eligible articles were then extracted and summarised using a predetermined table consisting of the following information: study design, study setting, sample size, age group, intervention types, treatment duration, control group, and outcome measures. Four authors (YNR, GR, R, and CAS) performed this process independently by reading the full-text of the articles.

### Quality Assessment

Four authors (YNR, GR, R, and CAS) critically appraised and determined the risks of bias for each article, which was confirmed by the fifth reviewer (SH) in case of discrepancies. Critical appraisal was conducted using the Joanna Briggs Institute checklists. Two checklists were used according to the study designs: randomised controlled trials and quasi-experimental studies. Each tool consisted of several “Yes,” “No,” “Unclear,” and “N/A” questions. Articles were considered to have lower risks of bias if more questions were answered “Yes.” Based on the researchers’ agreement, the articles were categorised into low (scored > 70%), medium (scored 50% to 70%), and high (scored < 50%) risks of bias. All articles were included regardless of the level of risk of bias in the analysis; however, articles with high risk of bias were flagged.

### Additional Resources

To enrich the discussion, relevant additional articles obtained from general Internet searches were added. Additional articles were subject to the same selection process, as well as data extraction and quality assessment procedures.

## Results

### Study Selection

Results of database search and additional sources are illustrated in Figure 1. This study initially retrieved 3,821 articles, with 461 articles from PubMed, 2,577 from ProQuest, and 783 from EBSCO. After eliminating duplicates, abstracts of 3,579 articles were screened, resulting in 27 articles for full-text review. The 27 studies were then assessed according to the selection criteria. Studies that had different focus in terms of population (one study), study design (seven studies), and types of intervention (five studies) were eliminated. One study with unavailable full-text and two with incorrect publication type were excluded. Four supplementary resources were added to this review, resulting in a total of 15 eligible studies.

### Study Characteristics

Fifteen studies were analysed in this review (Table 1). One study each was conducted in Iran (11), Sierra Leone (12), United Kingdom (13), Zambia (14), Kenya (15), and Indonesia (16), three in Malawi (17–19), four in India (20–23), and two multicenter studies in Kenya–Malawi (24) and China–Hongkong (25).

This review included four randomised controlled clinical trials (11, 13, 15, 20); one double-blind, parallel-group, randomised, controlled non-inferiority clinical trial (17); three double-blind randomised control trials (18, 19, 24); one randomised, triple-blind, controlled clinical non-inferiority trial (12); one nonblinded, parallel-group, cluster randomised, controlled, equivalence trial (14); one nonrandomised control trial (22); one multicenter, prospective, randomised, double-blinded study (21); and three randomised, controlled open-label trials (16, 23, 25).

Six studies were conducted in a community setting (11, 13, 15, 16, 19, 20), where health services are delivered to individuals, families, or community members with or without the involvement of primary health care services. Conversely, seven studies were performed in a hospital setting (12, 18, 21–25), and two were conducted in an outpatient programme setting (14, 17).

This review included children with various severity levels of malnutrition: children with SAM in nine studies (12–15, 17, 18, 20, 22, 24), MAM in one (19), mild or moderate malnutrition in two (11, 16), at risk of malnutrition (children with weight-to-height percentile within the 3rd and 15th percentiles) in two (21, 23), and with weight-for-height in the 15th percentile in one (25). Moreover, this review included three articles with low (12, 21, 24), 11 with medium (11, 13, 25, 15–20, 22, 23), and one with high risk of bias (14).

### Outcome Measurements

Two study outcomes were identified from the selected studies: nutrition-related and other outcomes (Tables 1 and S1). Nutrition-related outcomes included anthropometric and biochemical outcomes. Anthropometric outcomes included changes in weight or weight gain (11–14, 16, 17, 19–23), percentage of children achieving the intended weight (20), height increment (13, 21), changes in body mass index (BMI) (11, 21), changes in the weight-for-height z-score (WHZ) (11, 18, 19, 25), changes in the height-for-age z-score (HAZ) (13), changes in the weight-for-age z-score (WAZ) (25), changes in weight-for-height percentile (21), and changes in the mid-upper arm circumference (MUAC) (19, 21). Biochemical outcomes were changes in blood levels of docosahexaenoic acid (DHA) (15, 18), eicosapentaenoic acid (EPA), arachidonic acid (15, 18), and erythrocyte n-3 polyunsaturated fatty acids (PUFA) (15). The following outcomes related to daily food intake were also reported: energy (13, 21), protein (13), micronutrients (13, 25), and product intake, e.g. FSMP consumption (16) and improved appetite (13).

Other observed outcomes included stool characteristics (16), bone quality (25), recovery rate (11, 12, 14, 17–20), hospitalisation rate (12), time to stabilisation (24), time to graduation/time needed to achieve full recovery (12), length of stay (LOS) (14, 17, 22), remained SAM (12), prevalence of diarrhoea (11), prevalence of fever (11, 17), days with diarrhoea (24), days with enteral feeding (24), days with intravenous fluids (24), incidence of diarrhoea (25), incidence of respiratory infections (15, 23), adverse events (13, 15, 16, 21, 25), and mortality (12, 24).

### Interventions

Three types of FSMPs were reported: RUSFs (11, 19), RUTFs (12, 14, 15, 17, 18, 20, 22), ONS (13, 16, 21, 23, 25), and isocaloric modified F75 (24).

#### RUSFs

Two studies investigated the effectiveness of RUSFs in treating children with malnutrition. Azimi et al. (11) evaluated RUSFs containing soy protein isolate, whey protein, egg white, dates, vegetable oils, sugar, starch, and micronutrients. Children receiving 1–3 sachets (475 kcal, 15 g protein each) every two weeks for 8 weeks showed significantly higher recovery rate (92% vs. 12%), greater weight (1.44 ± 0.38 vs. 0.7 ± 0.32 kg, *P* < 0.001), BMI gains (1.2 ± 0.47 vs. 0.35 ± 0.33 kg/m^2^, *P* < 0.001), lower diarrhoea (12% vs. 28.6%, *P* = 0.01) and fever (16% vs. 36.7%, *P* = 0.05) prevalence than controls (11).

Stobaugh et al. (19) compared whey- and soy-based RUSFs. Whey-based RUSFs contained 18.7% whey permeate, 4.9% whey protein concentrate 80% (WPC80), peanut paste, sugar, palm oil, soy oil, emulsifier, and customised micronutrient premix. Conversely, soy-based RUSFs contained extruded soy flour, peanut paste, sugar, palm oil, soy oil, micronutrient premix, and dicalcium phosphate or calcium carbonate. For the same serving size (105.35 g), whey-based RUSFs contained fewer calories and proteins than soy-based RUSFs (516.35 vs. 559.52 calories and 11.42 vs. 17.06 g of protein). Despite lower energy and protein per serving, whey-based RUSFs yielded higher recovery in children with MAM (83.9% vs. 80.5%; RR 1.043; 95% CI: 1.003, 1.084; *P* < 0.04). Compared with the other group, the whey-based RUSF group exhibited higher weight gain during the first 2–4 weeks of therapy (*P* < 0.05), higher WHZ at the final measurement (*P* < 0.008), and greater WHZ increments (*P* < 0.02) (19).

#### RUTFs

Eight studies reviewed the effectiveness of modified RUTFs for children with malnutrition (12, 14,15, 17, 18, 20, 22, 24). Thakur et al. (22) reported that local RUTFs (L-RUTFs) made from groundnut (25%), milk powder (30%), sugar (30%), and vegetable oil (15%) promoted more significant weight gain (9.59 ± 3.39) than standard F-100 (5.41 ± 1.05). This result was corroborated by Jadhav et al. (20), who demonstrated that 8 weeks of intervention with 175 kcal/kg RUTF-1 (consisting of peanut butter, skimmed milk powder, powdered sugar, soya bean oil, and micronutrients) was more effective in promoting weight gain among children with SAM than standard nutrition therapy and locally prescribed diets. Local dietary prescription contained milk, sugar, oil, boiled eggs, banana, rice green gram porridge, vegetables, and jaggery. Thakur et al. (22) also reported a higher proportion of children recovering from SAM among those receiving L-RUTFs than those receiving locally prepared F-100 diet (82.8% vs. 19.3%, respectively).

Two studies evaluated the effects of flaxseed oil-containing RUTFs (F-RUTFs) (15), flaxseed oil-containing RUTFs with additional fish oil capsules (FFO-RUTFs) (15), and high-oleic acid RUTFs (HO-RUTFs) (18) on the plasma level of n-3 PUFA. Jones et al. (15) reported that children with lower plasma DHA levels at baseline experienced a more pronounced increase in DHA levels when provided with FFO-RUTFs; however, no significant changes were detected among those receiving F-RUTFs. Contrastingly, children with higher plasma DHA levels at baseline experienced a less significant increment when provided with FFO-RUTFs and substantial DHA level reduction when provided with F-RUTFs (15). Hsieh et al. (18) reported a significant increase in plasma PUFA levels and higher WHZ upon completion of intervention with HO-RUTFs. Compared with the standard RUTFs, the recovery rate of children who received HO-RUTFs was slightly lower (71% vs. 68%, respectively, *P* = 0.72) (18).

Three studies investigated the effectiveness of modified RUTFs with ingredients similar to the standard peanut-based or milk-based RUTFs. Bahwere et al. (17) examined the substitution of dried skimmed milk with whey protein concentrates 34% (WPC34) in RUTFs (WPC-RUTFs) and found comparable outcomes with standard peanut-based RUTFs (P-RUTFs), reporting recovery rates based on ITT analysis of 84.8% and 84.2%, respectively. Hendrixson et al. (12) found that an oat-based RUTFs (oat-RUTFs), which replaced peanuts with oat and excluded hydrogenated vegetable oil, yielded superior outcomes compared with standard RUTFs. Using the ITT analysis, Bahwere et al. (17) reported recovery rates of 84.8% and 84.8% in the WPC-RUTF and P-RUTF groups, respectively. Hendrixson et al. (12) also found that 56% of the children receiving oat-RUTFs and 45% of those receiving standard RUTFs achieved full recovery. Additionally, the graduation time was shorter in the oat-RUTF group than in the standard RUTF group, with 3.9 ± 1.8 vs. 4.5 ± 1.8 visits, respectively (*P* < 0.001) (12).

Bahwere et al. (17) also reported that LOS in the WPC-RUTF group was shorter than that in the P-RUTF group (32.6 [95% CI: 30.8, 34.5] vs. 34.5 [95% CI: 32.5, 36.5]) days using the ITT analysis and 34.2 (95% CI: 32.8, 36.8) vs. 35.8 (95% CI: 33.8, 36.3) days using the PP analysis. WPC-RUTFs resulted in faster recovery with 34.8 (95% CI: 32.8, 36.8) vs. 36.1 (95% CI: 33.8, 36.3) days and lower fever incidence (9.1%, 27/278) vs. (17%, 43/253) *(P* = 0.013) compared with P-RUTFs (17).

Irena et al. (14) reported that soy-maize-sorghum-based RUTFs (SMS-RUTFs) resulted in a lower recovery rate (60.8% and 81.8% vs. 53.3% and 77.9%, according to ITT and PP analyses, respectively) and lower weight gain (*P* = 0.007) than P-RUTF. SMS-RUTF therapy also resulted in longer LOS (35 [IQR: 28, 52] vs. 47 [IQR: 29, 70]) days than P-RUTFs (14). However, study findings should be interpreted with caution because of the high risk of bias stemming from its non-blinded study design, difference in baseline characteristics among groups, and a substantial number of patients with unknown final outcomes. Likewise, this study did not compare SMS-RUTFs and P-RUTFs in SAM management. Bandsma et al. (24) compared a lactose-free, reduced-carbohydrate isocaloric modified F75 (mF75) with the standard F75 in hospitalised children with SAM. They found no significant differences in stabilisation time (IRR 1.05 [0.91, 1.22]; *P* =0.48), biochemical indicators, other clinical outcomes, and risk of hospital mortality (24).

#### Oral Nutrition Supplement

Five studies examined the effect of ONS (13, 16, 21, 23, 25). Hubbard et al. (13) reported that compact-style oral nutrition supplement (cONS) improved energy and nutrient intake, with greater gains in weight, height, and HAZ compared with standard ONS (sONS). Compared with the sONS group, the study showed that cONS prescription resulted in significantly higher average total daily energy, protein, and key micronutrient intakes on day 2. After 28 days of intervention, the proportion of patients meeting their energy needs increased from 33% to 48% in the cONS group but remained unchanged in the sONS group. Additionally, the cONS group demonstrated substantial improvements in weight (*P* = 0.007), height (*P* < 0.001), and HAZ (*P* = 0.006) (13).

Khanna et al. (21) reported that after 90 days of intervention, both milk-based ONS with dietary counselling (ONS1 + DC) and lactose-free ONS with dietary counselling (ONS2 + DC) groups experienced a substantial increase in the weight-for-height percentile (*P* = 0.0086) and BMI (*P* < 0.05) compared with those receiving DC only. Higher height increments were also observed within the ONS group than within the DC group. MUAC improvement was also observed significantly in the ONS1 + DC group compared with that in the DC group (*P* < 0.05) (21). On the contrary, Devaera et al (16) reported that administering 400 mL/day of 1.5 kcal/mL ONS for 4 weeks vs. 600 mL/day of 1.0 kcal/mL ONS did not cause a difference in the total weight gain, recovery rate, stool characteristics, and gastrointestinal symptoms (16).

Ghosh et al. (23) and Sheng et al. (25) examined the benefit of ONS along with dietary counselling. Ghosh et al. (23) found that the mean weight gain of those receiving ONS with DC was almost twice that of those receiving DC only after 90 days of intervention (*P* < 0.0001). They also observed a remarkable increase in the WAZ score, BMI-for-age z-score, and incidence of upper respiratory tract infection (URTI) in the intervention group (23). Sheng et al. (25) found a similar effect of this intervention on days 30 (*P* = 0.047) and 90 (*P* = 0.021); however, the difference in the WHZ score increment between groups was not significant after 120 days of intervention (25).

### Outcome Comparison

This review showed that FSMP administration led to remarkable improvements in nutrition-related outcomes compared with standard dietary treatment in hospital, outpatient, and community settings. Improvements in anthropometric measures (weight gain, height gain, WHZ changes, and MUAC), biochemical indicators, and dietary intake were well observed, thus highlighting the efficacy of FSMPs in treating malnutrition.

Nine studies reported positive effects of FSMPs on daily weight gain, which included interventions with RUSFs (11, 19), RUTFs (12, 14, 17, 20, 22), and ONS (Figure 2) (16, 21). Daily weight gains were measured directly in seven studies (11, 12, 14, 17, 19, 20, 22) and estimated from the total weight gain in two studies (16, 21). RUSFs and RUTFs demonstrated significantly greater daily weight gain than standard diets. Moreover, RUSFs and RUTFs consistently outperformed standard nutritional therapy in improving weight among children with MAM and SAM (11, 20, 22). Likewise, milk-based or lactose-free ONS with both standard formula (1 kcal) and high-energy formula (1.5 kcal) were equally effective in increasing the weight of children with mild-to-moderate malnutrition (16, 21). Daily weight gain was the highest among those receiving RUTFs compared with RUSFs and ONS.

In community settings, Jadhav et al. (20), who modified the original RUTFs with peanut butter (25%), skimmed milk powder (24%), powdered sugar (28%), peanut oil soybeans (21%), micronutrients (1.6%), and emulsifiers (0.4%), reported the highest daily weight gain. In hospital settings, L-RUTFs made of peanuts (25%), powdered milk (30%), sugar (30%), and vegetable oil (15%) proved to be the most effective at achieving the highest daily weight gain (20, 22). In outpatient settings, the highest daily weight gain was using peanut milk-based RUTFs (14).

Five studies measured and reported daily (Figure 3A) and total (Figure 3B) height gains. Whey-based RUSFs (19) yielded the highest daily height gain in the community setting, and oat-RUTFs (12) in the hospital setting. The group administered FSMPs yielded higher height improvements than those who received normal diet and dietary counselling. Moreover, those receiving dietary counselling only appeared to achieve the lowest height gain (21).

Four studies examined the effect of FSMPs on WHZ (Figure 3C). Azimi et al. (11) reported that eight weeks of intervention with FSMPs yielded significant improvement in WHZ and resulted in a high proportion of children achieving normal nutrition status (11). Stobaugh et al. (19) determined that whey-based RUSF treatment was more effective in increasing WHZ after 12 weeks of treatment than soy-based RUSFs. However, treatment using ONS yielded inconclusive results in WHZ (16, 25).

Three studies reported significant daily MUAC gain after 12 weeks of intervention with whey- and soy-based RUSFs (19), oat-based and standard RUTFs (12), as well as milk-based and lactose-free ONS (Figure 3D) (21). Among these three studies, the highest daily MUAC gain was observed in the milk-based ONS and dietary counselling group (21).

Two studies evaluated changes in biochemical indicators such as blood levels of DHA, EPA, and arachidonic acid (AA) after treatment with RUTFs (15, 18). Jones et al. (15) reported that the 84-day provision of FFO-RUTFs increased blood DHA levels. AA levels increased significantly among those receiving standard RUTFs compared to the FFO-RUTF and F-RUTF groups. Similarly, Hsieh et al. (18) found that treatment with HO-RUTFs increased blood DHA and EPA levels in 2 weeks but reduced blood AA levels within 4 weeks.

The benefit of FSMPs was also determined by measuring the recovery rates of children with malnutrition (Figure 4A). Eight studies examined the effect of RUTFs (12, 14, 17, 18, 20), RUSFs (11, 19), and ONS (16). In community settings, Azimi et al. (11) reported the most notable outcome after 8 weeks of intervention with local food-based RUSFs. In the outpatient setting, Bahwere et al. (17) observed the highest recovery rate following 4 months of treatment with WPC-RUTFs. In the hospital setting, Hsieh et al. (18) found that treatment with RUTFs resulted in a 71% recovery rate. Among all FSMPs, the lowest recovery rate was observed among those receiving ONS for 29 days in a community setting, as reported by Devaera et al. (16). However, these studies used varied definitions of recovery rate and treatment duration; hence, careful interpretation is needed.

Three studies measured LOS as indicators of the efficacy of FSMPs; two were conducted in outpatient settings (14, 17) and one in a hospital setting (Figure 4B) (22). The shortest LOS was with WPC therapy in the community setting (17), whereas in the hospital setting, LOS was shorter in the RUTF group (13.04 days) than in the F-100 group (16.2 days) (22).

The prevalence, incidence, and frequency of diarrhoea were investigated in four studies. Azimi et al. (11) found that the prevalence of diarrhoea was lower in children receiving RUSFs than in those receiving normal diet (12% vs. 28.6%, *P* = 0.01). However, Hendrixson et al. (12) found no significant difference in the prevalence of diarrhoea between those receiving oat-RUTFs and s-RUTFs. Bahwere et al. (17) also reported the lack of a significant difference in diarrhoea frequency and duration between children receiving WPC-RUTF and P-RUTF. Moreover, Jones et al. (15) reported no difference in the incidence of diarrhoea when comparing s-RUTFs vs. F-RUTFs vs. FFO-RUTFs (*P* = 0.75) (15).

Other common illnesses that were used as indicators of FSMP efficacy included fever, cough, and URTI. RUSFs and WPC-RUTF were associated with lower fever prevalence (11, 17). However, Hendrixson et al. (12) found no difference in the prevalence of fever between those receiving oat-RUTFs and s-RUTFs. Conversely, Bahwere et al. (17) reported a lower frequency of cough in the WPC-RUTF group than in the P-RUTF group after 1 week of treatment. Hendrixson et al. (12) also found lower cough prevalence in the oat-RUTF treatment group than in the s-RUTF group. Furthermore, fewer URTI cases were documented among those in the ONS + DC group than in the DC group (23). Jones et al. (15) also reported lower URTI rates in the s-RUTF than in the F-RUTF and FFO-RUTF groups; however, the difference was not significant.

Three studies evaluated the adverse events of FSMP therapy. In the study by Ghosh et al. (23), adverse events were documented in 18.9% of children in the ONS+DC group and 21.9% in the DC group. Khanna et al. (21) reported adverse events in 24.3% of children in the ONS1 + DC group, 30.8% in the ONS2 + DC group, and 18.7% in the DC group. Sheng et al. (25) reported 81.8% of adverse events in the NC + NS group and 71.1% in the NC group. The most common adverse events included URTI, gastrointestinal tract issues, fever, and diarrhoea (21, 23, 25).

### Cost Analysis

Four studies documented the economic value of FSMPs in treating malnutrition. Three studies compared the cost between modified RUTF and standard RUTF (12, 14, 17), and one study examined the cost of RUSF-modified recipes (19).

Milk powder accounted for more than half of the cost of standard P-RUTF; thus, replacing it with a more affordable protein source would result in cheaper RUTF. Bahwere et al. (17) suggested that replacing milk powder with WPC34 in WPC-RUTF would reduce the cost by 25% to 33%. This study found that using the modified recipe yielded nearly similar outcomes in weight gain, recovery rate, organoleptic acceptability, and tolerance with P-RUTF (17). Irena et al. (14) also showed that replacing milk powder with locally available grain and pulses in SMS-RUTFs could reduce the ingredients cost by 33% (USD810 per tonne). However, children receiving SMS-RUTFs had lower recovery rates and weight gain than those receiving P-RUTF (14).

Hendrixson et al. (12) reported that oat-RUTF cost 15% less than standard P-RUTF (USD1.68 vs. 1.98/kg). Oat-RUTF was proven to be superior to s-RUTFs because it promoted better growth outcomes and higher graduation rates. Children receiving oat-RUTF also had lower risks of adverse events. However, considering that colder climatic conditions are more suitable for oat cultivation, oat-RUTF production will be more feasible and cost-effective in certain regions such as North America, Ethiopia, or South Africa (12).

Additionally, Stobaugh et al. (19) found that whey-based RUSFs (USD3.13/kg) were more expensive than soy-based RUSFs (USD2.78/kg). To treat a child weighing 7 kg, approximately 3 kg of RUSFs was needed until recovery, which resulted in the cost difference between the two products as ~USD1.49 per child treated, or USD1.36 per child recovered. However, treatment with whey-based RUSFs yielded better outcomes than soy-based RUSFs in terms of recovery rate, MUAC changes, weight gain, and WHZ (19).

## Discussion

To be considered FSMPs, dietary prescriptions should be specifically formulated for patient dietary management and administered under medical supervision through exclusive or partial feeding (9, 26). FSMPs should be developed for patients with specific dietary needs, including those unable to meet their nutrition requirements through regular diet or other specialised foods because of limited digestive capacity or other medical issues (9, 26). The Food and Agriculture Organization and the World Health Organization define RUTF (27) as a high-energy FSMP containing essential nutrients and protein needed for treating children aged 6 to 59 months with SAM without any medical complications related to their appetite. It must be crushable, soft, and ready to eat without any further preparation (27). Conversely, the British Specialist Nutrition Association defines ONS as a specialised food designed to meet the nutritional needs of individuals living with temporary or permanent medical issues that prevent them from obtaining adequate nutrition from regular foods and are at risk of malnutrition. ONS may be used as a partial or complete substitute for the usual diet and should be administered exclusively under medical supervision, commonly available as ready-to-drink beverages (28). However, no guidelines regulating RUSF as an FSMP are available, although it complies with the FSMP definition. RUSFs are a fortified lipid-based paste/spread specifically formulated for 2 to 3 months of intervention as a part of MAM treatment for children aged ≥ 6 months. RUSFs should be consumed directly from the package without dilution, mixing, or cooking (29).

This systematic review examined studies on children under five with malnutrition requiring nutrition interventions either in hospital or community settings. The results revealed that FSMPs significantly improved weight, height, WHZ, and BMI in comparison with dietary counselling, placebo, or the usual diet. Moreover, children who received FSMPs had higher recovery rates from malnutrition.

First, weight gain was the most commonly measured indicator of FSMP efficacy across the included studies. The highest daily weight gain was recorded in the L-RUTF group treated in the hospital and community setting and in the P-RUTF group in the outpatient setting. The lowest daily weight gain was reported in the ONS group. Rimbawan et al. (30) also documented that RUTF resulted in higher weight gain compared with other interventions, such as high-calorie cereal meals, supplementary foods based on maize and soy flour, and the F-100 formula. Of the nine studies that observed weight gain, only two achieved the standard of 5 g/kg/day of weight gain (20, 22, 31), which might be linked to water retention due to oedema, fluid shifts, diarrhoea, and hydration status (17, 32).

Second, linear growth or height was also reported as an outcome of FSMP interventions (33–36). Two studies found that the RUSF and ONS groups experienced more significant daily height gain than the standard diet group (11, 13, 21). Significant improvements were observed following 8 weeks of RUSF, 4 weeks of cONS, and 90 days of either milk-based or lactose-free ONS. Hendrixson et al. (12) reported that 12 weeks of oat-RUTF in a hospital setting led to a similar result. RUSF, specifically designed to support recovery from malnutrition, yielded the highest WHZ increments (11, 37, 38), whereas ONS yielded the least changes in WHZ (16). Nevertheless, ONS-based treatment remained superior in promoting growth among children with malnutrition than normal diet, even with longer intervention duration (39).

Third, an increase in MUAC was reported as a treatment outcome of all types of FSMPs directly or indirectly (40–43). In the hospital setting, ONS1 (milk-based) + DC improved MUAC significantly compared with RUTF (12) and DC (21) alone. In the community setting, whey-based RUSFs yielded higher daily MUAC changes than standard RUSFs (19).

Fourth, FSMP efficacy was also assessed by recovery rate. The highest recovery rates were observed among those receiving RUSFs (11), whereas the lowest was found in the ONS group (16). In the outpatient setting, the highest recovery rate was reported in the WPC-RUTF group (17), and the lowest was found among the SMS-RUTF groups (14). However, most studies did not achieve the minimum recovery rate threshold, likely due to the short intervention duration and comorbidities such as oedema and pneumonia (31, 44). In the community setting, the recovery rate of malnutrition might be influenced by family socioeconomic status and availability of micronutrient supplementation and deworming programmes (45). Nevertheless, previous studies have shown the superiority of FSMP to standard nutrition therapy and normal diet in promoting recovery rate (30, 31, 39).

Fifth, few studies have reported LOS as an efficacy measure of FSMPs. In the hospital setting (22), LOS was shorter in the L-RUTF group than in the F-100 group, consistent with findings in children with SAM (34). In the outpatient setting, LOS was not different between children in the WPC-RUTF and SMS-RUTF groups (14, 17). Both studies used P-RUTF instead of placebo as control, which were corroborated by previous reviews (31, 46).

Finally, modifying FSMP formulas with local ingredients has been a global practice to reduce costs and increase acceptability (47). However, only a few studies have documented the cost of administering FSMPs to children with malnutrition. This review included four studies that compared the production cost of standard RUTF or RUSFs and their alternatives. The results were inconclusive to support the benefit of changing the FSMP formula with local ingredients. For example, Kohlmann et al. (46) reported that RUTF modification, which reduced the cost by 14% compared with the standard recipe, exhibited lower efficacy in treating SAM and MAM in Ghana. Likewise, although substituting soy with whey in RUSFs led to better clinical outcomes, the production costs were higher (19).

To the best of our knowledge, no studies have evaluated the health-economic effect of prescribing FSMPs for children with malnutrition. Studies reporting costs merely explored the production fee of FSMPs without considering other medical and nonmedical costs and without comparing the benefits of doing such interventions. Previous studies have not reached consensus on whether FSMPs are cost-effective in treating malnutrition in children. In the USA, the administration of ONS to hospitalised paediatric patients was found to be cost-effective, resulting in a 9.7% reduction in hospitalisation expenses (USD16,552 vs. USD18,320; difference of USD1,768 [95% CI: USD1,924, USD1,612]) and a 14.8% decrease in LOS (6.4 vs. 7.5 days; reduction of 1.1 [95% CI: 0.2, 2.4] days) (48). In contrast, supplementing general food distribution with RUSFs to children with non-acute malnutrition in urban Chad was not cost-effective. This intervention increased the total cost by 23%, leading to an extra expenditure of EUR374 per child, along with an added cost of EUR1,083 per prevented episode of diarrhoea and EUR3,627 for each prevented case of anaemia (49).

### Limitations

Several limitations should be considered while interpreting the findings of this review. First, among the 15 studies, only three are categorised as having minimal risks of bias. Second, some studies used different defining variables, such as discharge criteria to determine recovery, duration of intervention, study settings, and initial conditions. Lastly, the health-economic effect of each FSMP intervention was not evaluated because of data unavailability. Therefore, whether FSMPs and their alternatives are cost-effective in treating malnutrition in children cannot be confirmed.

## Conclusions

Most studies included in this review concluded that prescribing FSMPs to treat malnutrition in hospital, outpatient, and community settings led to better growth outcomes than counselling, placebo, or usual diet. However, concerning the heterogeneity of studies analysed in this review, future research must be conducted based on agreement on intervention design and study boundaries to increase the comparability of findings.

Unfortunately, the health-economic effect of FSMPs could not be confirmed in this review. Therefore, more studies are required to understand the direct and indirect health-economic consequences of providing FSMPs to treat malnutrition in children. Although the clinical benefit of FSMP administration has been well documented, information on the economic values of the intervention is important to make better decisions in health at the macro level.

## Supplementary Material

**Table S1:** Other outcomes and findings

Author Country	Other outcomes	Other findings	
Azimi et al. ( 11) Iran	Prevalence of diarrhoea and fever	Children who received RUSF had lower prevalence of diarrhoea (12% vs. 28.6%, *P* = 0.01) and marginally lower fever rate (16% vs. 36.7%, *P* = 0.05)	
Bahwere et al. ( 17) Malawi	Frequency of fever, cough, and diarrhoea; length of stay (LOS)	Children with WPC-RUTF had lower frequency of fever than the P-RUTF; *P* = 0.013 at week 1 No significant difference in frequency of cough between groups( *P* = 0.082) at week 1 No significant difference in frequency of diarrhoea at week 1( *P* = 0.227) and duration (*P* = 0.478) between groups LOS difference between groups −1.6 days (95% CI: −4.6, 1.4 days) in PP analysis and −1.9 days (95% CI: −4.6, 0.8 days) for ITT analysis	
Bandsma et al. ( 24) Kenya and Malawi	Adverse events	No difference in number of children who died before stabilisation phase between groups (*P* = 0.84), total days with diarrhoea (*P* = 0.27), enteral therapy (*P* = 0.42) or intravenous fluids ( *P* = 0.19) 38% developed diarrhoea in hospital	
Hendrixson et al. ( 12) Sierra Leone	Time to graduation, rates of weight gain, rates of MUAC gain, prevalence of diarrhoea	Time to graduation was shorter in the oat-RUTF group; 3.9 ± 1.8 vs. 4.5 ± 1.8 visits (*P* < 0.001) Rates of weight gain were higher in the oat-RUTF group; 3.4 ± 2.7 vs. 2.5 ± 2.3 g/kg/d (*P* < 0.001) Rates of MUAC gain were higher in the oat-RUTF group; 0.24 ± 0.24 mm/ day vs. 0.18 ± 0.23 mm/day (*P* < 0.001) Length gain was higher in the oat-RUTF group; 2.23±1.55 vs. 2.09 ± 1.55 cm No significant difference in prevalence of diarrhoea, fever, and cough between groups	
Hsieh et al. ( 18) Malawi	Recovery rate from SAM, WHZ	No significant difference in terms of recovery rate from SAM (defined as MUAC > 12.4 cm without oedema at week 12) between groups (*P =* 0.72) Children receiving HO-RUTF had greater WHZ upon completion of treatment (*P* = 0.02) There was significant difference in the level of arachidonic acid between groups (*P* < 0.009)	
Hubbard et al. ( 13) United Kingdom	weight, height, appetite, adverse events	Growth indicators improved in both groups, but results in the cONS group were significant [weight *P =* 0.007), height **(***P* < 0.001), and height *z*-score (*P =* 0.006)] More patients with cONS reported improved appetite compared to the sONS group (*P =* 0.018) Nine adverse events were reported, four were not related to the product (cONS *n* = 2, sONS *n* = 2), three were gastrointestinal symptoms possibly related to the product (cONS *n* = 2, sONS *n* = 1), and two were vomiting, which related to the cONS	
Irena et al. ( 14) Zambia	Weight gain, oedema, LOS	The SMS-RUTF group had lower weight gain than the P-RUTF [2.2 (1.9, 2.5) vs. 3.2 (2.9, 3.5) g/kg/d, *P =* 0.007] with oedema (*P =* 0.018) and non-oedema (*P =* 0.091) cases No difference in median of LOS between groups (*P =* 0.494). For those who were discharged as recovered, the median LOS was 35 days (IQR, 28 to 52 days) for P-RUTF group and 47 days (IQR, 29 to 70) for SMS-RUTF group (*P* < 0.001) Between the study arm and age group, an interaction occurred (*P <* 0.001 for ITT analyses and 0.0683 for PP analyses). The ARDs were −10.0 (−17.7 to −2.3) % for ITT (*P* = 0.013) and −4.7 (−10.0 to 0.7) for PP (*P* = 0.083) analyses for the < 24 months age group and 2.1 (−10.3, 14.6) % for ITT (*P* = 0.726) and −0.6 (−16.1, 14.5) for PP (*P* = 0.939) for the 24 months age group	
Jadhav et al. ( 26) India	-	-	
Jones et al. ( 15) Kenya	Adverse events, incidence of diarrhoea, URTI, and acceptability	More children in the F-RUTF and FFO-RUTF groups experienced upper respiratory tract infection (URTI) and vomiting No difference in incidence of diarrhoea between s-RUTF, F-RUTF, and FFO-RUTF groups (*P* = 0.75) There was significant difference in incidence of URTI between s-RUTF, F-RUTF and FFO-RUTF groups (*P* = 0.08) RUTF with added short chain n-3 PUFA and fish oil capsules was acceptable to participants	
Stobaugh et al. ( 19) Malawi	Daily MUAC gain, weight gain, length gain, WHZ change, recovery time	Compared to the soy RUSF group, the whey RUSF group had higher: Daily MUAC gain (0.26 ± 0.27 vs. 0.22 ± 0.28 mm/day, *P* = 0.0025) Daily weight gain (2.95 ± 2.04 vs. 2.79 ± 2.16 g/kg/d, *P* = 0.11) Length gain (0.30 ± 0.28 vs. 0.29 ± 0.29 mm/day, *P* = 0.18) WHZ change (0.77 ± 0.62 vs. 0.70 ± 0.66, *P* = 0.012) Whey RUSF group had slower recovery time than soy RUSF group (29.3 ± 19.0 vs. 30.4 ± 20.1, *P* = 0.22)	
Thakur et al. ( 22) India	LOS	The LOS in the L-RUTF group was significantly shorter than the F100 group (13.04 ± 0.16 days vs. 16.2 ± 4.73 days) ( *P* < 0.0001; 95% CI: −4.502, 1.818)	
Khanna et al.( 21) India	Weight increment, changes in height, BMI changes, MUAC changes, energy intake, adverse events	Weight gain differed significantly between ONS1 + DC vs. DC only group (*P* = 0.0012) and between the ONS2 + DC vs. DC only group (*P* = 0.0012) The ONS1 + DC and ONS2 + DC groups showed higher but not statistically significant changes in height compared to the DC only group There were significant differences in BMI changes between ONS1 + DC vs. DC only group (*P* = 0.0185) and between ONS2 + DC vs. DC only group (*P* = 0.0064) MUAC changes differed significantly*P* (= 0.0418) between the ONS1 + DC vs. DC only group The mean of energy intake improved significantly for both ONS1 + DC and ONS2 + DC groups Adverse events were reported in the ONS1 + DC, ONS2 + DC and DC only groups with 24.3%, 30.8%, and 18.7%, respectively. The most frequently reported adverse events were respiratory and gastrointestinal tract events	
Devaera et al.( 16) Indonesia	Daily weight gain	Daily weight gain between intervention and control groups were (g/kg/day) 1.2 ± 1.1 vs. 1.3 ± 1.2, *P* = 0.60	
Ghosh et al. ( 23) India	Incidence of URTI, adverse events	The incidence rate of URTI was 2.01 times more frequent in the control group than in the intervention group (*P* = 0.0361), using the ITT population Adverse events were reported in both groups	
Sheng et al. ( 25) China and Hong Kong	Intake of calcium, phosphorus, iron, zinc, vitamin A, C, D, E, and B6; incidence of diarrhoea, upper and lower respiratory tract infections	The NC + NS group had significantly higher intake of calcium, phosphorus, iron, zinc, vitamin A, C, D, E, and B6, at day 60 and 120 (*P* < 0.001) The *z*-scores for quantitative ultrasound (QUS) bone measures were not significantly different between groups at day 120 The incidence of diarrhoea, upper and lower respiratory tract infections did not differ between groups. Two children in the NC + NS group experienced moderate constipation or mild diarrhoea, which considered to be study-related	

## Figures and Tables

**Figure 1:**
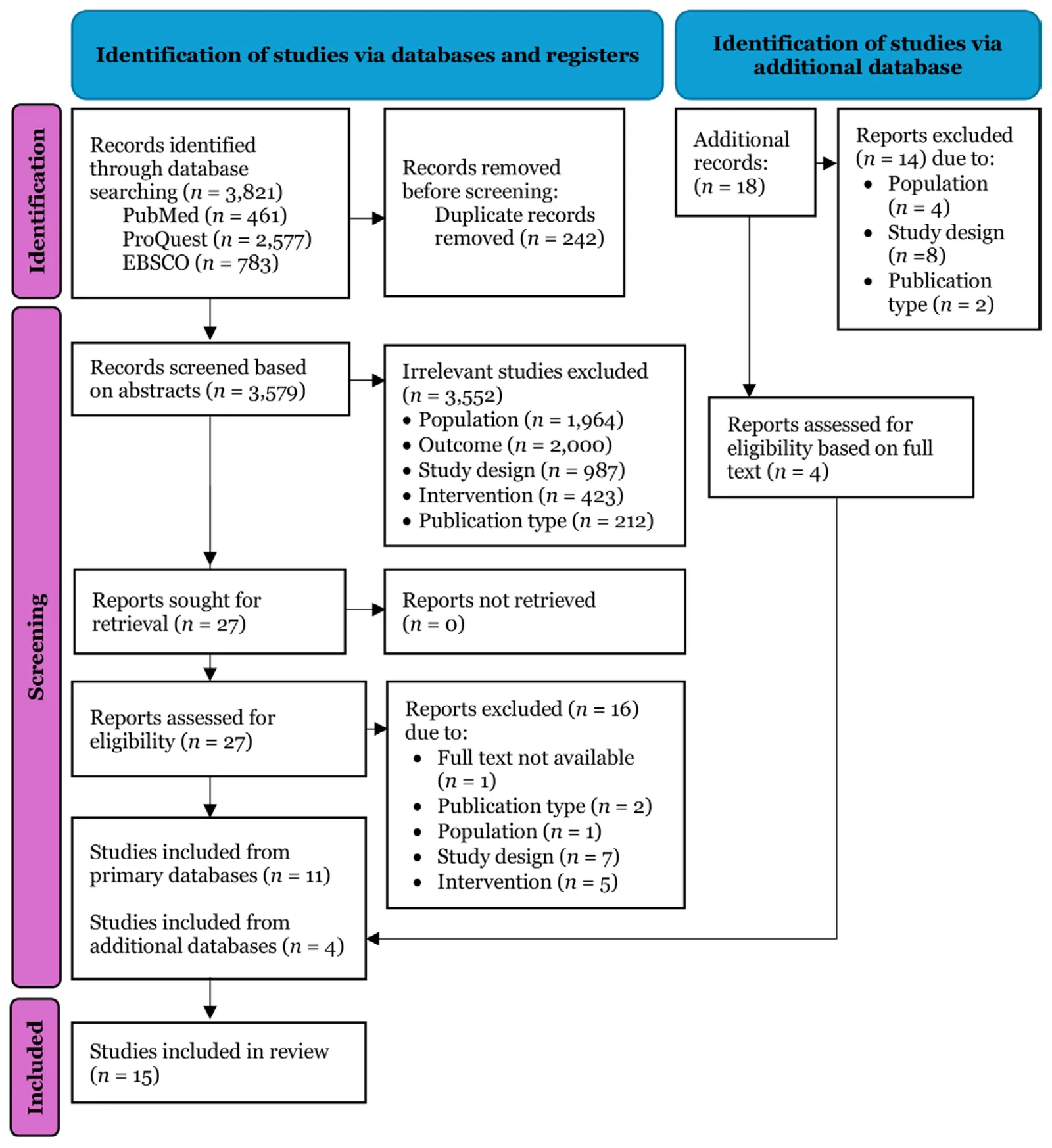
Data collection process

**Figure 2:**
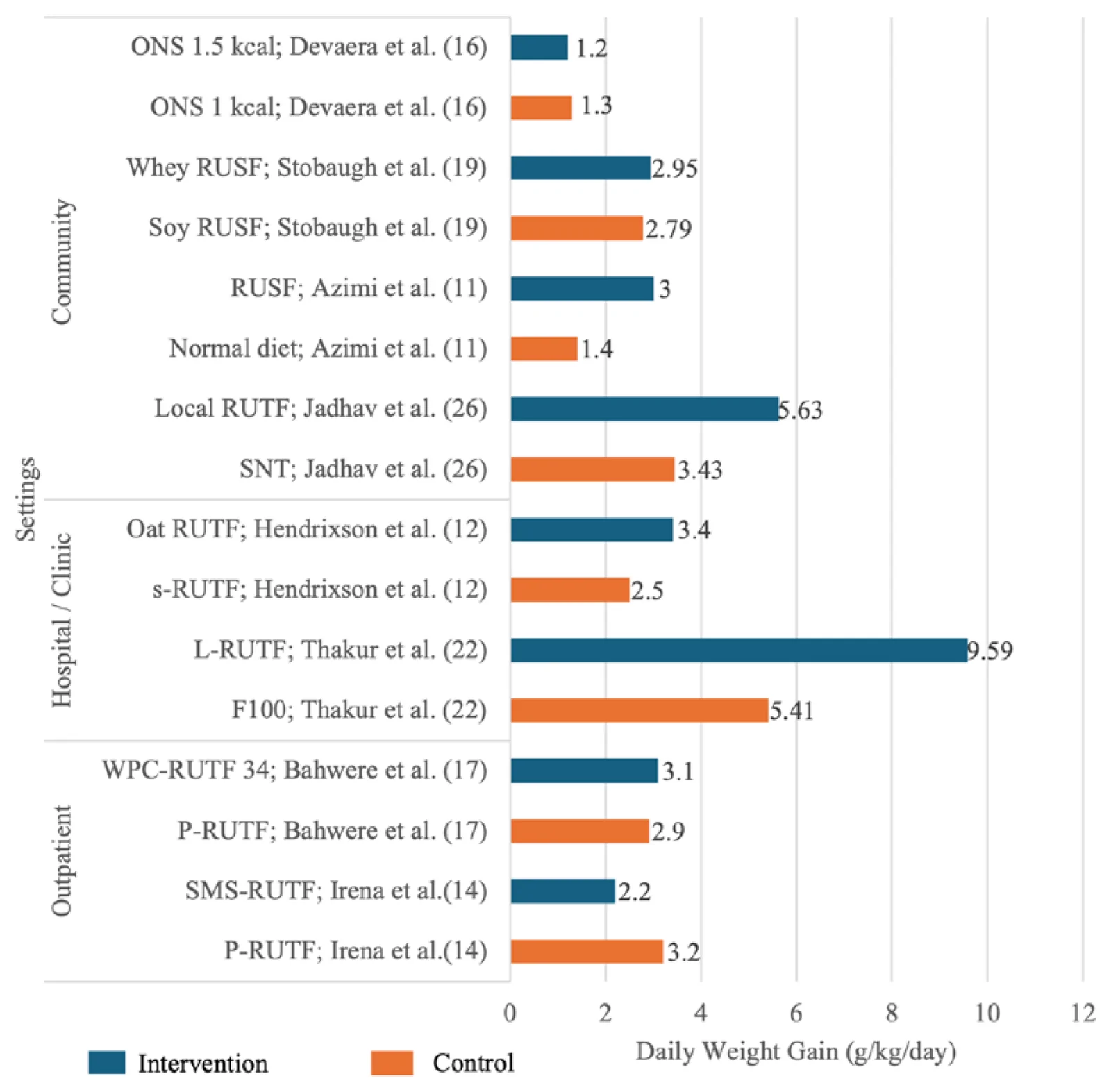
Daily weight gain achieved from administering the FSMPs

**Figure 3:**
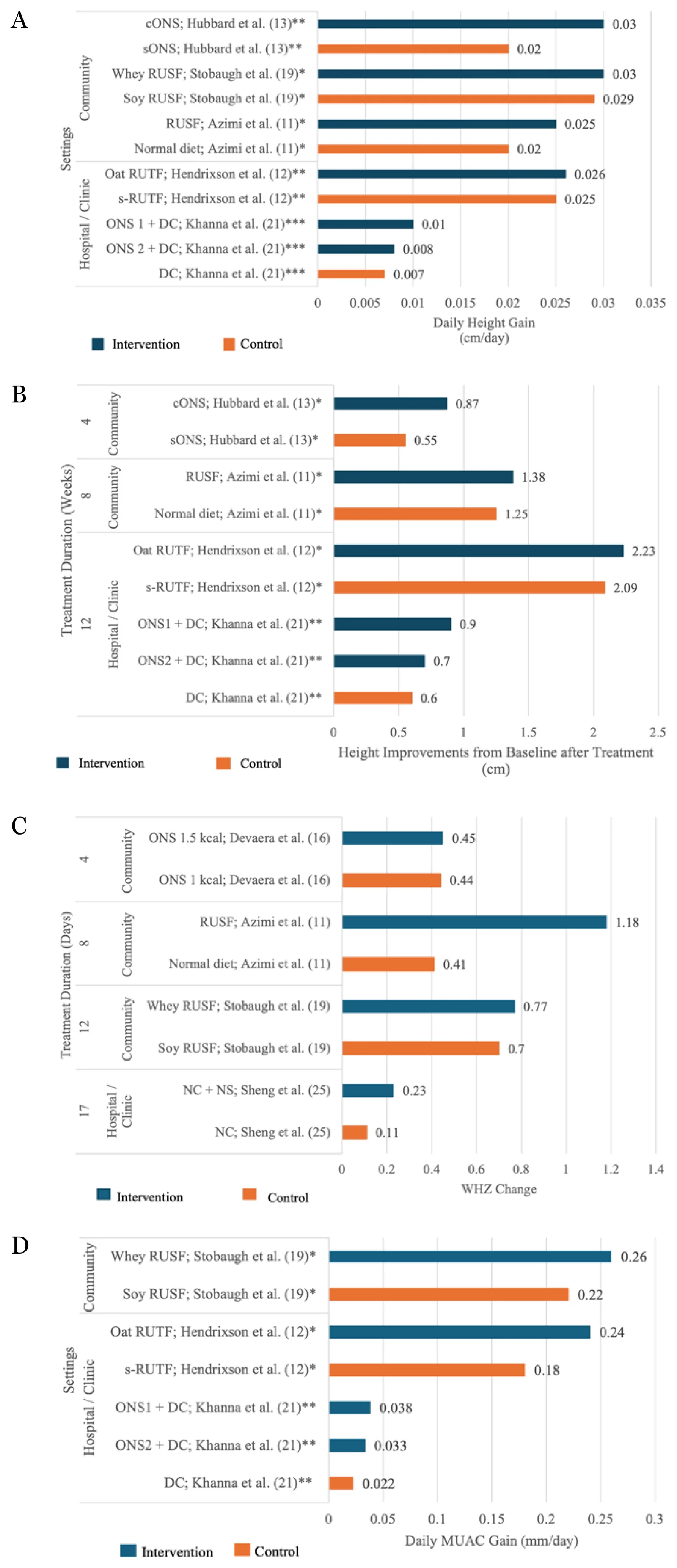
Anthropometric outcomes achieved from administering the FSMPs A: *Mean daily height gain; **Mean daily height gain estimated from total; ***Median daily height gain estimated from total; B and D: *Mean; **Median

**Figure 4:**
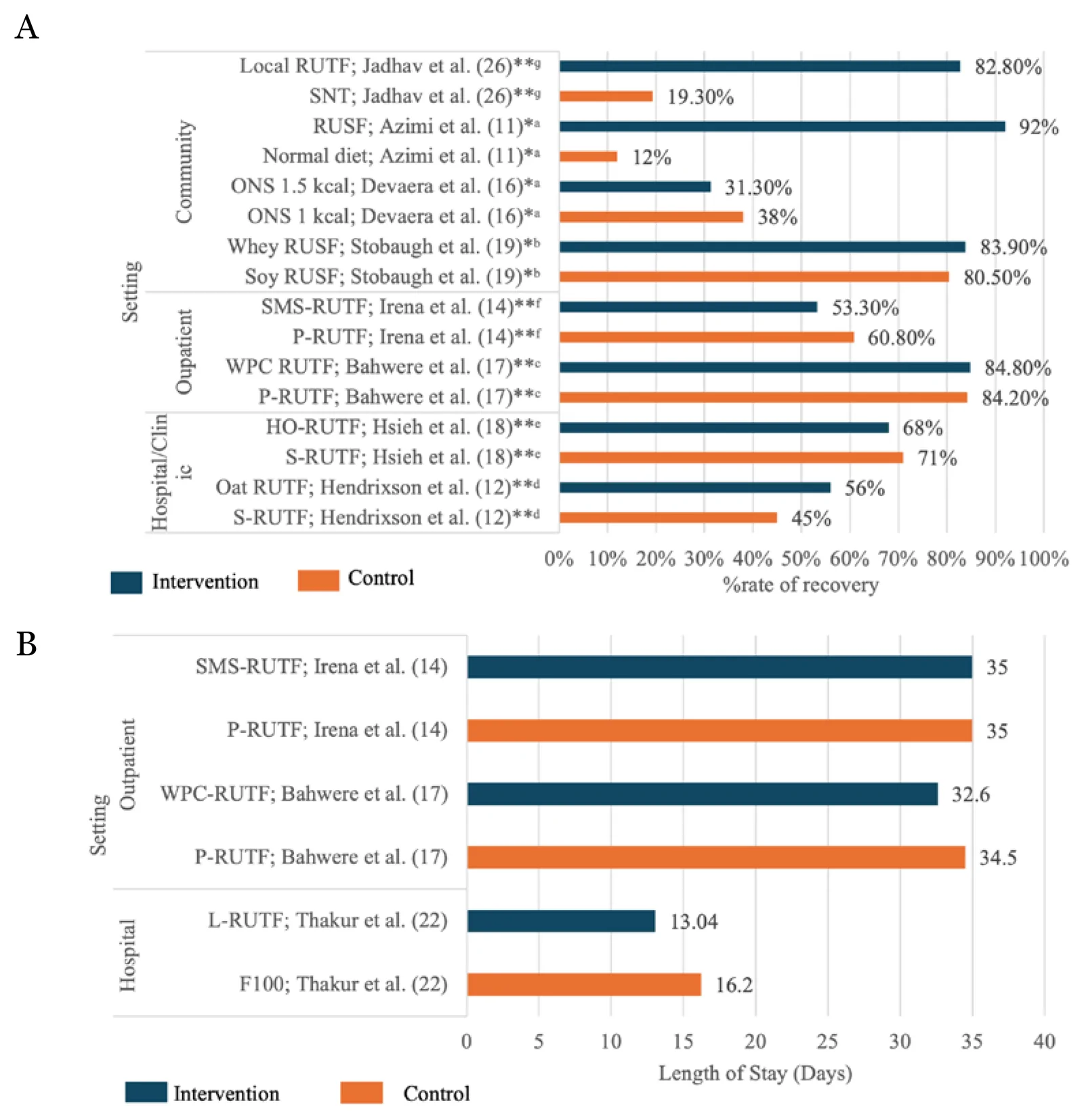
Rate of recovery and length of stay of children between the intervention and control groups A: *Recovery from Mild or Moderate Acute Malnutrition; **Recovery from Severe Acute Malnutrition Definition of recovery: ^a^WHZ > −1; ^b^MUAC ≥ 12.5 cm without peripheral oedema; ^c^weight gain of at least 15%, MUAC > 11.0 cm, no medical complication, no bilateral pitting oedema, and a minimum of 1 month treatment (for children admitted with MUAC < 11.0 cm) or no bilateral pitting oedema, clinically well and MUAC > 11.0 cm (for children admitted with bilateral pitting oedema); ^d^MUAC > 12.4 cm, WLZ ≥ −2; ^e^MUAC > 12.4 cm without oedema; ^f^weight gain of at least 18%, MUAC > 11.0 cm, no medical complication, and no bilateral pitting oedema (for children admitted with MUAC < 11.0 cm) or no bilateral pitting oedema, clinically well, and MUAC > 11.0 cm (for children admitted with bilateral pitting oedema); ^g^weight for height *z*-score > −3 SD or MUAC > 115 mm

**Table 1:** Study intervention, outcomes, and findings

Author Country	Description of study design and intervention	Main outcomes	Main findings
Azimi et al. (11) Iran	Children were randomly assigned to receive either 1–3 sachets of RUSF or normal diet Duration: 8 weeks Design: Randomised controlled clinical trial Setting: Health centres in urban area Sample: 100 children (24 to 59 months old) with mild or moderate malnutrition	Growth indicators: weight and height, weight-for-height z-score (WHZ), and body mass index (BMI)	Children who received RUSF had a significant increase in: Weight (1.44 ± 0.38 vs. 0.7 ± 0.32 kg, *P* < 0.001) BMI (1.2 ± 0.47 vs. 0.35 ± 0.33 kg/m^2^, *P* < 0.001) Daily weight gain at week 4 (2.6 ± 0.8 vs. 0.95 ± 0.7 g/kg/day, *P* < 0.001) Daily weight gain at week 8 (3.0 ± 1.5 vs. 1.4 ± 0.2 g/kg/day, *P* = 0.013) Daily height gain at week 4 (0.025 ± 0.011 vs. 0.02 ± 0.01 cm/d, *P* = 0.027) WHZ gain (1.18 ± 0.41 vs. 0.41 ± 0.31, *P* < 0.001) Rate of recovery (AHR: 16.3; 95% CI: 6.1, 43.3; *P* = 0.001) 92% of children in the RUSF group and 12% in the control group have reached WHZ > −1
Bahwere et al. (17) Malawi	Children were evenly randomised to WPC-RUTF (intervention) or P-RUTF (active control) 34% whey protein concentrates were used in WPC-RUTF to replace dried skimmed milk. P-RUTF contained peanut paste, milk powder, oil, sugar, minerals and vitamins Duration: 1 week to 4 months Design: Double-blind, randomised, controlled non-inferiority trial with parallel-group Setting: Outpatient treatment programme (OTP) Sample: 600 children (6 to 59 months old) with severe acute malnutrition (SAM)	Mean of weight gain, recovery rate	WPC-RUTF was not inferior to P-RUTF in terms of recovery rate [difference = 0.5% (95% CI: −2.7, 3.7) in per protocol (PP) analysis and 0.6% (95% CI: −5.2, 6.3) in intention to treat (ITT) analysis] and mean of weight gain [0.2 (−0.5; 0.9) for both analyses] Average weight gain was 3.1 g/kg/day for WPC-RUTF and 2.9 g/kg/day for P-RUTF group In the WPC-RUTF group, the recovery rate, mortality rate and defaulter rate for ITT analyses were 84.8% (257/303), 1.6% (5/303) and 12.2% (37/303), respectively In the P-RUTF group, the recovery rate, mortality rate and defaulter rate for ITT analyses were 84.2%, 0.7% and 12.2%, respectively
Bandsma et al. (24) Kenya and Malawi	Hospitalised severely malnourished children were randomised to standard F75 or isocaloric, lactose-free modified F75 with low carbohydrate Duration: 5 days Design: Double-blind randomised control trial Setting: Hospitals Sample: 843 children (6 months to 13 years old) with SAM	Time to stabilisation	Median time to stabilisation was 3 days (IQR 2 to 5 days) [IRR 1.05(0.91, 1.22) *P* = 0.48]
Hendrixson et al. (12) Sierra Leone	Participants were given a 2-week ration of RUTF (approximately 150 kcal/kg/day). Children returned fortnightly for follow up, and additional RUTF was distributed for those who remained wasted for a maximum of 12 weeks Duration: 2 weeks, with a maximum of 12 weeks follow up Design: Randomised, triple-blind, controlled clinical non-inferiority trial Setting: Health clinics in rural areas Sample: 1406 children (6 to 59 months old) with SAM	Time to graduation, moderate acute malnutrition (MAM) and SAM recovery, died/ transferred to inpatient care, or defaulted, determined by weight-for-length z-score (WLZ) and mid-upper arm circumference (MUAC)	There was significant difference in time to graduation (MUAC > 12, 4 cm, WLZ ≥ −2) between oat-RUTF and s-RUTF groups (difference 10.6%; 95% CI: 5.4, 15.8) Death rates, hospitalisation, and rates of treatment failure were significantly different between groups (difference 6.2%; 95% CI: 2.3, 10.0, *P* = 0.001)
Hsieh et al. (18) Malawi	High-oleic acid RUTF (HO-RUTF) vs. standard RUTF. All participants received RUTF that provided about 175 kcal/kg/d (735 kJ/kg/d) Duration: 2 weeks of ration with 4 to 12 weeks of follow up Design: Prospective, randomised, double-blind clinical effectiveness trial Setting: Clinics in rural areas Sample: 141 children (6 to 59 months old) with SAM	Change in plasma docosahexaenoic acid (DHA) and eicosapentaenoic acid (EPA) after 4 weeks	During the first 4 weeks, the HO-RUTF experienced significant changes in: DHA level (*P =* 0.04) EPA level (*P* < 0.001)
Hubbard et al. (13) United Kingdom	Participants were given daily energy-dense, low volume paediatric-specific ready-made, cONS 300 kcal/125 mL orally Duration: 28 days Design: Prospective, interventional, parallel randomised controlled trial Setting: Community-based paediatric care Sample: 51 children (1 to 12 years old) with faltering growth and/or requiring ONS to meet nutrition requirements	Nutrient intake	cONS resulted in significantly higher mean of total daily energy intake (+531 kcal/day), protein (+10.1 g/ day), and key micronutrients than the sONS’ group at day 2 and improved appetite The percentage of patients meeting their energy requirements increased with cONS (33% to 48%), but did not change with sONS (ITT and PP)
Irena et al. (14) Zambia	All children received a 5-day course of amoxicillin, a single 100 mg dose of mebendazole, a 1-week ration of RUTF, and nutrition education. The RUTF ration was calculated to provide 200 kcal/ kg/day, repeated for 1 week with similar dosage until discharge. The intervention group received SMS-RUTF while the control group received P-RUTF Duration: 1 week of ration with additional weekly rations until discharge (no maximum number of weeks specified) Design: Non-blinded, parallel-group, cluster randomised, controlled, equivalence trial Setting: Outpatient clinics Sample: 1,927 children (6 to 59 months old) with SAM	Recovery rate	Based on the ITT and PP analyses, the recovery rate in the SMS-RUTF group was lower than the P-RUTF group (53.3% and 60.8% for the ITT and 77.9% and 81.8% for PP analyses). The adjusted risk difference and 95% CI were −7.6% (−14.9, 0.6%) and −3.5% (−9.6, 2.7%) for ITT (*P =* 0.034) and PP (*P* = 0.257), respectively
Jadhav et al. (26) India	The intervention group received RUTF-1 (MNT) at 175 kcal/kg/ day. Caregivers received nutrition counselling, and the children shifted to homemade diet after eight weeks Duration: 8 weeks and followed up for 4 months Design: Prospective randomised controlled trial Setting: Hospitals/clinics and community settings Sample: 1,105 children (6 to 60 months old) with SAM	Mean of weight gain rate Proportion of children achieving target weight Recovery from SAM status	Mean of weight gain rate was significantly higher in children receiving RUTF-1 (MNT) compared to the standard nutrition therapy (SNT) group (5.63 g/kg/day vs. 3.43 g/kg/day, *P* < 0.05) Proportion of children achieving the target weight was 60.4% in the RUTF-1 (MNT) group and 47.8% in the SNT group At 8 weeks, 82.8% of children in the RUTF-1 (MNT) group recovered from SAM compared to 19.3% in control
Jones et al. (15) Kenya	Children were grouped into standard RUTF (S-RUTF), flaxseed oil-containing RUTF (F-RUTF), and flaxseed oil-containing RUTF plus fish oil capsules (FFO-RUTF) groups Duration: 84 days Design: Randomised controlled trial Setting: Rural communities Sample: 60 children (6 to 50 months old) with SAM	Erythrocyte n-3 polyunsaturated fatty acid (PUFA) level at day 84	In the FFO-RUTF group, children with low baseline DHA had more significant increase compared to those with higher baseline DHA, unlike in the S-RUTF and F-RUTF groups. There was significant decrease in DHA level among children who had higher DHA level at baseline (*P* = 0.045 overall, and *P* = 0.025 between the S-RUTF and F-RUTF groups)
Stobaugh et al. (19) Malawi	Children were randomly assigned to receive daily dose of ~75 kcal/kg/d of soy RUSF or whey RUSF Duration: 12 weeks Design: Randomised, double-blind controlled clinical Setting: Rural communities Sample: 2,259 children (6 to 59 months old) with MAM	The proportion of children who recovered from MAM within 12 weeks of treatment	More children recovered from MAM in the whey RUSF group than in the soy RUSF group (RR: 1.043; 95% CI: 1.003, 1.084; *P* < 0.04) The risk difference for recovery rate between the whey RUSF and the soy RUSF group was 3.4% (95% CI: 0.3, 6.6)
Thakur et al. (22) India	Children received either 12 g/kg/ day L-RUTF or 60 mL/kg/day of F100 in 4 packages (approximately 60 kcal/kg/day of therapeutic food and 60 kcal/kg/day of family food) Duration: During rehabilitation phase Design: Non-RCT Setting: Hospital Sample: 118 children (6 to 60 months old) with SAM	Rate of weight gain (g/kg/day)	The rate of weight gain in the L-RUTF group was 9.59 ± 3.39 g/ kg/d, while in the F100 group was 5.41 ± 1.05 g/kg/d Significant difference in weight gain in the L-RUTF group (*P* < 0.0001; 95% CI: 3.174, 5.186)
Khanna et al. (21) India	Oral Nutrition Supplement 1 (ONS1/milk-based) + Dietary Counselling (DC) ONS2 (lactose-free) + DC DC only Duration: 90 days Design: Multi-centre, prospective, randomised, double-blinded study Setting: Hospitals and clinics in urban or semi-urban areas Sample: 321 children (24 to 48 months old) at risk of malnutrition (weight-for-height percentile 3rd to 15th)	Change in weight-for-height percentile	Changes in weight-for-height percentile were more significant in the ONS1 + DC group and ONS2 + DC group, compared to the DC only group (*P* = 0.0086) No significant difference between ONS1 and ONS2 treatment groups (*P* = 0.935) at day 90
Devaera et al. (16) Indonesia	The intervention group received 400 mL/day of 1.5 kcal/mL ONS, and the control group received 600 mL/day of 1.0 kcal/mL ONS Duration: 4 weeks Design: A parallel, randomised, controlled open-label trial Setting: Community-based outreach clinics Sample: 110 children (3 to 6 years old) with mild-to-moderate malnutrition	Daily product consumption Body weight Tolerance and dietary intake from solid food	Both groups had similar compliance and acceptance levels to the products; only 16.7% of children in the intervention group and 14% in the control group did not like the products (*P* = 0.71) There were no significant differences in total weight gain, daily weight gain, and the percentage of children reached WHZ score of > −1 There were no significant differences in stool characteristics and gastrointestinal symptoms
Ghosh et al. (23) India	Children in the intervention group (ONS+DC) received: One serving (224 mL) of ONS for children 24 to 48 months Two servings (448 mL) of ONS children 48 to 72 months Control group: DC only Duration: 90 days Design: A prospective, randomised, controlled, open-label, parallel-group, multicenter study Setting: Clinics and hospitals Sample: 255 children (24 to 72 months) at risk of malnutrition (weight-for-height percentile 3rd to 15th)	Changes in growth (weight-for-age z-score, height-for-age z-score, BMI-for-age *z*-score)	By day 90, the weight gain in the intervention group was almost double the control group (*P* < 0.0001)
Sheng et al. (25) China and Hong Kong	Children randomly assigned to nutrition counselling (NC) alone or nutrition counselling and nutritional milk supplement (NC + NS) groups Duration: 120 days Design: Multicenter, open-label, randomised, controlled trial Setting: Hospitals Sample: 153 children (30 to 60 months old) whose height-for-weight is in the 25th percentile	Changes in WHZ	The differences in WHZ changes between groups were not significant. The adjusted mean (SE) of WHZ was 0.23 (0.06) in the NC + NS group and 0.11 (0.06) in the NC group (between-group difference, 0.12 [95% CI: −0.04, 0.29]; *P* = 0.137) WHZ increased significantly in the NC + NS group at day 30 (*P* = 0.047) and 90 (*P* = 0.021) and also for the entire study period (between-group difference, 0.13 [95% CI: 0.01, 0.25]; *P* = 0.029) The adjusted mean (SE) of WHZ in the NC + NS group were significantly higher at day 90 (0.03 [0.03] vs. 0.14 [0.03]; *P* = 0.025) and for the entire study period (between-group difference, 0.08 [95% CI: 0.00, 0.16], *P* = 0.046)
